# Spatial-Temporal Dynamic Evolution of Land Deformation Driven by Hydrological Signals around Chaohu Lake

**DOI:** 10.3390/s24041198

**Published:** 2024-02-12

**Authors:** Tingye Tao, Ju Dai, Zichen Song, Shuiping Li, Xiaochuan Qu, Yongchao Zhu, Zhenxuan Li, Mingming Zhu

**Affiliations:** College of Civil Engineering, Hefei University of Technology, Hefei 230009, China; taotingye@hfut.edu.cn (T.T.); songzc@mail.hfut.edu.cn (Z.S.); lishuiping@hfut.edu.cn (S.L.); qqxxcc@hfut.edu.cn (X.Q.); yczhu@hfut.edu.cn (Y.Z.); zxli2019@hfut.edu.cn (Z.L.); zhumingming@mail.hfut.edu.cn (M.Z.)

**Keywords:** hydrological signals, heavy rainfall, GNSS, water level, environmental load

## Abstract

The frequent occurrence of extreme climate events has a significant impact on people’s lives. Heavy rainfall can lead to an increase of regional Terrestrial Water Storage (TWS), which will cause land subsidence due to the influence of hydrological load. At present, regional TWS is mostly obtained from Gravity Recovery and Climate Experiment (GRACE) data, but the method has limitations for small areas. This paper used water level and flow data as hydrological signals to study the land subsidence caused by heavy rainfall in the Chaohu Lake area of East China (June 2016–August 2016). Pearson’s correlation coefficient was used to study the interconnection between water resource changes and Global Navigation Satellites System (GNSS) vertical displacement. Meanwhile, to address the reliability of the research results, combined with the Coefficient of determination method, the research findings were validated by using different institutional models. The results showed that: (1) During heavy rainfall, the vertical displacement caused by atmospheric load was larger than non-tidal oceanic load, and the influence trends of the two were opposite. (2) The rapidly increasing hydrologic load in the Chaohu Lake area resulted in greater subsidence displacement at the closer CORS station (CHCH station) than the more distant CORS station (LALA station). The Pearson correlation coefficients between the vertical displacement and water level were as high as −0.80 and −0.64, respectively. The phenomenon confirmed the elastic deformation principle of disc load. (3) Although there was a systematic bias between the different environmental load deformation models, the deformation trends were generally consistent with the GNSS monitoring results. The average Coefficients of determination between the different models and the GNSS results were 0.63 and 0.77, respectively. The results demonstrated the effectiveness of GNSS in monitoring short-term hydrological load. This study reveals the spatial-temporal evolution of land deformation during heavy rainfall around Chaohu Lake, which is of reference significance for water resource management and infrastructure maintenance in this area.

## 1. Introduction

Chaohu Lake is one of the famous five freshwater lakes in China, with a variety of service functions such as water supply, fishery and tourism. Chaohu Lake basin is located on the left bank of the lower reaches of the Yangtze River (main stream area) and belongs to the subtropical humid monsoon climate zone. The average annual precipitation is about 1100 mm, with heavy rainfall mainly concentrated in June-August, accounting for about 50% of the average annual precipitation. Therefore, the rise and fall process of floods in the Chaohu Lake area is caused by heavy rainfall, water conservancy projects and other factors, which inevitably affect the nearby land deformation, damage buildings and even threaten the lives of residents [1,2]. 

With the development of technology, the traditional surface deformation monitoring methods are gradually replaced by Global Navigation Satellites System (GNSS), and the comprehensive use of GNSS technology can provide users with all-weather, real-time information on precise positioning accuracy, velocity and time, which has become one of the main technical means for detecting the deformation of the earth’s surface [3,4,5,6]. Meanwhile, the load deformation can be measured by numerous other methods, such as InSAR [7] and VLBI [8], etc. The Gravity Recovery and Climate Experiment (GRACE) satellite was developed by National Aeronautics and Space Administration (NASA) and German Aerospace Center (DLR). Compared to traditional hydrological signals monitoring, GRACE satellite [9,10,11] can provide high-precision, high spatial-temporal resolution hydrological data [12], which has been widely used around the world to estimate changes in Terrestrial Water Storage (TWS). Furthermore, some other monitoring data [13,14] (e.g., groundwater level data, regional precipitation data, etc.) can be used to carry out a quantitative analysis of groundwater storage changes and surface subsidence, especially for studying surface subsidence driven by regional hydrological signals in changing environments.

GNSS, GRACE and other multi-source data have been used to invert the surface subsidence during extreme weather. Zhang et al. [15] combined GRACE and GPS to retrieve the changes in groundwater storage during two drought periods in California and found that groundwater deficit was the main cause of surface subsidence. Nespoli et al. [16,17,18] found that hydrologic loads were negatively correlated with climate-driving factors such as snow cover (represented by snow depth) and precipitation in arid regions by using water level and surface displacement data. Bendick & Birhanu et al. [19] utilized the data of 16 GNSS base stations in Ethiopia and Eritrea in the drought variable regions to distinguish monsoon rains from other different seasonal loading models. Han et al. [20] used 120 GNSS base stations in Australia to capture trends in surface deformation during the record-breaking precipitation period of 2010–2011 and the following drought period. Mouyen et al. [21] found that typhoon transit accompanied by heavy rainfall caused significant surface deformation by combining surface air pressure data, nontidal sea level variations model, and rainfall data. Yao et al. [22] investigated the regional surface displacement and spatial-temporal evolution of hydrological water resources during typhoons by GPS and environmental load-induced deformation data. Luan et al. [23] based on Sentinel -1B SAR data for the Shouguang flood in 2018 and conducted related disaster research analysis. Song et al. [24] based on InSAR data, carried out a study on the flood-driven surface deformation monitoring in Nanchang, and the research can provide certain support for the safety monitoring of water conservancy projects and disaster early warning.

To address the challenge of land deformation driven by the regional water cycle, a number of scientific experiments have been carried out. Based on previous research results, in order to avoid the inapplicability of GRACE data in a small range [14,25], water level and flow data were used for hydrological signals in the paper. Using the Pearson correlation coefficient method, it was found that the change of Chaohu Lake water level during the heavy rainfall had an obvious effect on the displacement of CORS stations, which revealed the spatial-temporal dynamic evolution of land deformation in the Chaohu Lake area and confirmed the elastic deformation principle of disc load. At the same time, the environmental loading model data provided by International Mass Loading Service (IMLS) and GeoForschungsZentrum (GFZ) [26,27] was used to verify the research findings, the models can reflect the vertical deformation caused by the change of surface hydrological load around Chaohu Lake. The Coefficient of determination showed that the deformation trends of the models were generally consistent with the GNSS monitoring results. The research further demonstrated the effectiveness of GNSS in monitoring short-term hydrological load. This study can be used to evaluate the influence of water level on land deformation in the Chaohu Lake area. It has a certain scientific reference for the management and utilization of groundwater resources, which also has practical application value for the construction and maintenance of infrastructure in the Chaohu Lake area.

## 2. Materials and Methods

### 2.1. Materials

#### 2.1.1. Theoretical Support—Disc Load Elastic Deformation

When surface water, snow, ice and atmosphere act on the solid earth, the earth’s surface will produce load deformation, the use of Green’s function can establish the relationship between the load mass and deformation [28]. By first calculating the load love number, then calculating the load Green’s function, and convolving the load change with the Green’s function, the displacement of the target point can be obtained. Load deformation contains horizontal and vertical directions, vertical displacement deformation is more sensitive to the load source, and its load deformation is about 2–3 times of the horizontal load deformation [29].

The surface mass load deformation is the sum of these unit points mass load deformation, so the unit point source function is the basis for calculating the load deformation. When the unit point mass load is uniformly distributed at the poles, the surface density satisfies:

(1) $$\sigma \left(\phi \right)=\left\{\begin{array}{c} \sigma_{0}\;\;\phi \leq \beta \\ 0\;\;\;\phi >\beta \end{array}\right.$$

where σ(ϕ) is the global surface density, $\sigma_{0}$ is the density of the disc, β is the pole angle, and ϕ is the cosine latitude.

Since the total mass is 1, integrating the above equation:

(2) $$a^{2}\int_{0}^{2\pi }d\lambda \int_{0}^{\pi }\sigma \left(\phi \right)sin\left(\phi \right)d\phi =1$$

where a is the radius of the Earth and λ is the longitude. According to the symmetry of the Earth model, Equation (3) is obtained.

(3) $$2\pi a^{2}\sigma_{0}\int_{0}^{\beta }sin\left(\phi \right)d\phi =2\pi a^{2}\sigma_{0}(1-cos\beta )$$

Combining Equations (2) and (3).

(4) $$\sigma_{0}=\frac{1}{2\pi a^{2}(1-cos\beta )}$$

Therefore, when covering the ideal Earth’s surface with a uniform disc of mass, α is the angular radius, the equivalent water height h denotes its mass and $\rho_{w}$ is the density of water. The mass of the disc is obtained according to Equation (5).

(5) $$M=2\pi a^{2}\left(1-cos\alpha \right)h\rho_{w}$$

When the angle to the center of the disc is θ, the load deformation acting on the unit area can be calculated.

(6) $$\gamma \left(\theta \right)=\sum_{n=0}^{\infty }\Gamma_{n}P_{n}\left(cos\theta \right)$$

where $P_{n}$ is the Legendre polynomial, the derivation of the $\Gamma_{n}$ function is shown in Equations (7) and (8).

(7) $$\Gamma_{n}=\frac{1}{2}\left[P_{n-1}\left(cos\alpha \right)-P_{n+1}\left(cos\alpha \right)\right]\;\;\;n>0$$

(8) $$\Gamma_{0}=\frac{1}{2}(1-cos\alpha )$$

The Green’s function of the load-induced vertical displacement is shown in Equation (9) [30].

(9) $$S_{up}=\sum_{n=0}^{\infty }h_{n}\Gamma_{n}\frac{4\pi Ga}{g\left(2n+1\right)}P_{n}(cos\theta )$$

where θ is the angular radius to the center of the disc, G is Newton’s gravitational constant, a is the radius of the Earth, $h_{n}$ is the loading love number, g is the gravitational acceleration.

By using the above equations, vertical displacements due to surface mass changes on a regional scale can be calculated by spatial convolution:

(10) $$U_{total}=\sum_{Q\in \Omega }U\left(\theta ,\alpha \right)\Delta \sigma \left(Q\right)$$

where Q is the disc, Ω is the sum of all mass discs in the region, and Δσ(Q) is the change in mass at the load point.

**Figure 1:**
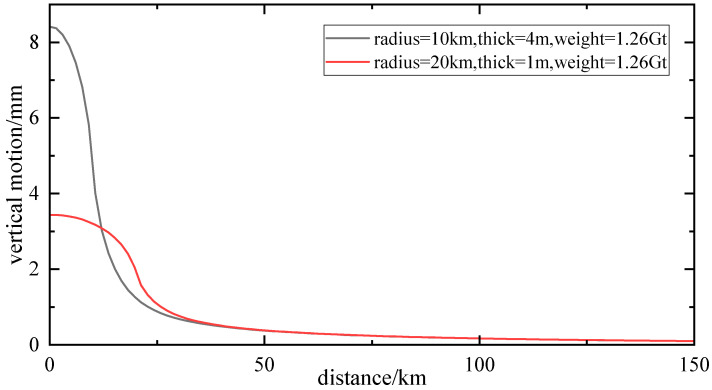
Schematic diagram of elastic deformation response to vertical displacement.

Suppose discs of the same mass, with different radii and thicknesses, which are placed on the Earth’s surface. It can be seen from Figure 1 that the maximum vertical displacement is induced at the center of the disc, with effects of 8.4 mm and 3.4 mm, respectively, and the response of the vertical deformation becomes smaller and smaller as the distance from the observation point to the center of the disc increases. When the distance to the center of the disc and the radius of the disc are equal, the load response is only half of the response of the center, when the distance to the center of the disc is about 5 times the radius, the load response basically disappears. The results show that: the vertical displacement of the Earth’s surface caused by the disc is related to the distance between the load point and the observation point. It decreases with the increase of the distance between the two points, the vertical deformation of the Earth’s surface caused by the region near the center of the disc is very obvious, and the influence of the more distant region gradually tends to zero. The influence of the vertical displacement of the surface caused by the mass load source is limited [31].

#### 2.1.2. GNSS Data

**Figure 2:**
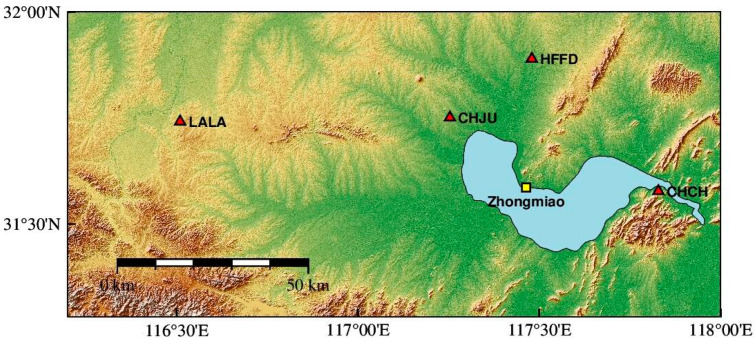
Distribution of AHCORS stations and water level observation station around Chaohu Lake.

The area of Chaohu Lake is about 780 km^2^, which is equivalent to a disc with a radius of 12.76 km. Based on the elastic deformation principle of disc load and data integrity, observation files of four reference stations (CHCH, CHJU, HFFD, and LALA) from June 2016 to August 2016 were studied. The data was provided by Anhui Provincial Bureau of Surveying and Mapping. And the stations are located in the vicinity of Chaohu Lake, as shown in Figure 2 (red triangles). CHCH station is located about 1 km due east of the Chaohu Lake, and the other three stations are farther away from the lake. Stations CHJU, HFFD and LALA are about 8 km, 21 km and 75 km away from Chaohu Lake, respectively.

In order to improve the accuracy of the solution, the analysis center of the International GNSS Service (IGS) and the global data center obtained the data files, ephemeris files and clock difference files of nine historical contemporaneous IGS stations (BJFS, DAEJ, IRKT, KIT3, LHAZ, POL2, TCMS, URUM, and WUHN) outside the study area, so as to form a regional network with the AHCORS stations. The data processing is divided into two main stages: (1) Solve the observed data from AHCORS and IGS stations using GAMIT to obtain the single-day relaxation solution, and the data-solving strategy is shown in Table 1. (2) Utilize the global h-file released by GLOBK in conjunction with SOPAC to perform the net leveling process, so as to obtain the coordinate time series of the AHCORS reference stations in the framework of ITRF2008.

#### 2.1.3. Water Level and Flow Data

The hydrological data of Chaohu Lake was provided by the Hefei Hydrology Comprehensive Service System, and the water level adopted the telemetry data from the representative station of Chaohu Lake, Zhongmiao Station, as shown in Figure 2 (yellow square). The time interval of the raw water level data was counted every hour. In order to facilitate the acquisition of data, the MATLAB tool was used to obtain the daily water level data of the station. Combined with the viewpoint of literature [32], entering the main flood season, the downstream water level of Chaohu Gate was higher than that of the upstream, and the gate was in a closed state. On June 20, the water level of Chaohu Lake was 9.27 m, 0.86 m higher than that of the same period of the year, and the water level of Chaohu Lake did not rise or fall significantly 20 days before the main flood season. After June 20, due to the strong rainfall in Chaohu Gate area, the water level of Chaohu Lake showed a rising trend. From July 1, it rose rapidly and exceeded the warning water level (10.50 m). After July 2, the water level above the gate was higher than that below the gate, and Chaohu Gate was opened for drainage. The average flow rate was about 525 m^3^/s, and the maximum amount of gate was up to 832 m^3^/s. On July 5, Chaohu Lake water level over the flood protection level (12.50 m), on July 9, the water level rose to the highest point of the flood (12.77 m), and then slowly fell back.

#### 2.1.4. Environmental Load-Induced Deformation Data

The AHCORS stations coordinate time series computed from GNSS observation files mainly contain the effects of two types of crustal non-tectonic deformation [33]. The first category is tidal deformation, including oceanic, polar and solid tides, and the IERS03 and FES2004 models have been selected to correct for tidal deformation when solved by using the GAMIT/GLOBK software. The second category is the surface loading variations caused by the mass migration of the atmosphere and each state of water in the hydrosphere of the Earth’s surface, which mainly includes Atmospheric Loading (ATML), Non-Tidal Oceanic Loading (NTOL) and Hydrological Loading (HYDL), etc. [34,35,36].

The MERRA2 model from IMLS is obtained by Global Simulation and Assimilation Office (GMAO) of NASA. The model provides estimates of global land surface conditions from 1980 to the present. MERRA2 calculates ATML and HYDL using spherical harmonic conversion and loading love numbers based on observed data products. MPIOM06 model, provided by the Musk-Planck Institute for Meteorology, calculates NTOL based on Green’s function. The LSDM model from GFZ utilizes data including snow cover and glacier runoff, and obtains HYDL by convolution based on Green’s function. Besides, the model data provided by IMLS has a higher spatial resolution than GFZ. So the model data of atmospheric and non-tidal oceanic loads used in this paper was provided by IMLS, the terrestrial hydrological loads were obtained by IMLS and GFZ together. The information of the products of the two is shown in Table 2.

### 2.2. Methods

#### 2.2.1. Pearson Correlation Coefficient (PCC)

Pearson’s correlation coefficient method was proposed by the British mathematician Karl Pearson in the late 19th century, and it was applied to measure the degree of correlation between two variables. It is now becoming a widespread statistical tool that has been used to analyze data in various fields [37].

If there is a correlation between the hydrologic load and vertical land deformation? We obtain the periodicity of the time series by two different spatial technologies. There are two possibilities: (1) The period of two time series is actually the same cycle. Both of them are caused by the same physical factors. But two different periodical signals are revealed, due to the different analyzing methods used or the limitations of the resolution and positioning accuracy. (2) There is a correlation between the physical factors that cause the different periodical signals. Therefore, the correlation between the GNSS time series and the hydrological signals can be used to determine whether they contain the same periodic term, or whether they contain periodic vibrations generated by the same physical factors. On the other hand, if the correlation is not significant, further exploration is needed. The calculation equation has the advantages of being simple to compute and easy to interpret as shown in Equation (11) [38]. The value range of Pearson correlation coefficient is shown in Table 3.

(11) $$PCC=\frac{\sum_{i=1}^{n}\left(\left(x_{i}-\bar{x}\right)y_{i}-\bar{y}\right)}{\sqrt{\sum_{i=1}^{n}{\left(x_{i}-\bar{x}\right)}^{2}\sqrt{\sum_{i=1}^{n}{\left(y_{i}-\bar{y}\right)}^{2}}}}$$

where the $x_{i}$ and $y_{i}$ represent two different variables, respectively, and the $\bar{x}$ and $\bar{y}$ denote the mean value of data.

#### 2.2.2. Coefficient of Determination (R^2^)

The concept of Coefficient of determination was first proposed by Sewall Wright in 1921. Its principle was based on the least square regression analysis method, which was used as a measure of the fit between the regression model and the observed data [39]. At present, the Coefficient of determination is generally used to validate the simulation results of hydrological models, which can explain the variation degree according to the independent variables. Assuming that the time series of the observation results is $X=\left(x_{1},x_{2},\cdots x_{N}\right)$, the time series of the simulation results is $S=\left(s_{1},s_{2},\cdots s_{N}\right)$, and the $\bar{x}$ and $\bar{s}$ denote the mean value of data, the consistency analysis is performed by the Equation (12). Coefficient of determination takes the value of 0 to 1, it close to 1 indicates that the model is of good quality and the model is credible, and the simulation results are generally consistent with the observation results. The calculation equation is as follows [40]:

(12) $$R^{2}=\frac{\sum_{i=1}^{N}\left(s_{i}-\bar{s}\right)}{\sqrt{\sum_{i=1}^{N}{\left(x_{i}-\bar{x}\right)}^{2}}}$$

#### 2.2.3. Framework of the Research Methods

**Figure 3:**
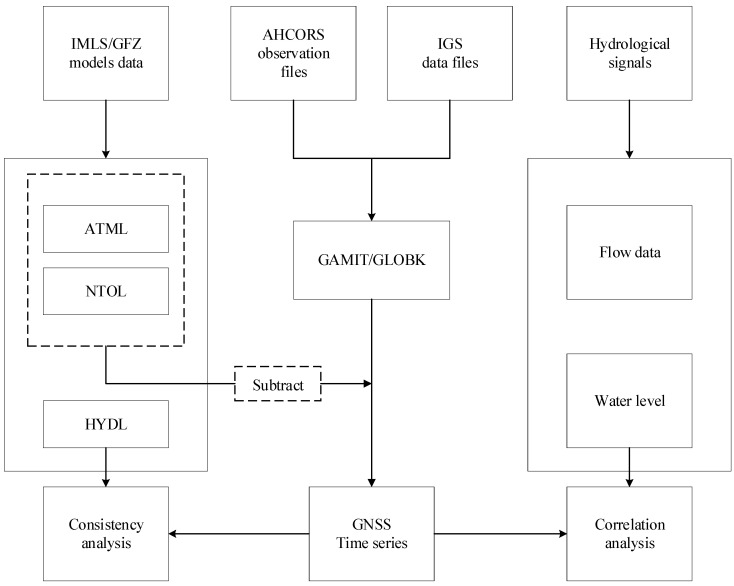
The flow chart of the research.

The paper was supported by the elastic deformation principle of disc load, and combining the above data and methods to obtain conclusions of the study. The framework of the research methods can be divided into the following steps. The process of the research methods is shown in Figure 3.

1: We utilized data such as the AHCORS observation files and historical contemporaneous IGS data files, applying the GAMIT/GLOBK to obtain the raw coordinate time series of the AHCORS reference stations in the framework of ITRF2008. In the process of using GAMIT/GLBOK software, the relevant parameters were selected to improve the accuracy of time series solution.

2: Since the raw GNSS time series contains the total environmental loads, the load-induced deformation model data of IMLS was used for correction. By subtracting the atmospheric and non-tidal oceanic loading deformations from the raw GNSS vertical displacement, the hydrological loading deformations in the GNSS vertical displacement can be accurately extracted [41].

3: Due to the rough observation of the GRACE satellite, which is not applicable to small areas. To avoid this problem, the water level and flow data of Chaohu Lake were used as hydrological signals. Water level data was used to analyze the vertical deformation of surface hydrological load and the flow data was combined to understand the rainstorm process of Chaohu Lake.

4: In order to obtain the spatial-temporal dynamic evolution of land deformation driven by hydrological signals around Chaohu Lake, the Pearson’s correlation coefficient was used to study the interconnection between water level change and GNSS vertical displacement. Based on the research results, the elastic deformation principle of disc load was confirmed.

5: The environmental load model data provided by IMLS and GFZ was used to calculate HYDL, and the consistency between HYDL and GNSS results was analyzed by using the Coefficient of decision. The results demonstrated the effectiveness of GNSS in monitoring short-term hydrological load and increased the reliability of the research results of the paper.

## 3. Results

In this section, the environmental load model data of IMLS was used to analyze the vertical deformation process of AHCORS stations under atmospheric and non-tidal oceanic loads during heavy rainfall. The study found that atmospheric load had a greater effect on vertical land deformation than non-tidal oceanic load. Then, Pearson correlation coefficient method was used to explain the influence of hydrological signals on surface subsidence, and it was found that the change of water level in the Chaohu Lake area played a major role in the change of surface elevation, which revealed the spatial-temporal dynamic evolution of land deformation driven by hydrological signals around Chaohu Lake and confirmed the elastic deformation principle of disc load. Finally, based on the Coefficient of determination method, the study concluded that the model data provided by IMLS and GFZ were generally consistent with GNSS monitoring results, which verified the influence of hydrological load on surface deformation around Chaohu Lake.

### 3.1. Effect of Atmospheric and Non-Tidal Oceanic Loads on Vertical Land Deformation

**Figure 4:**
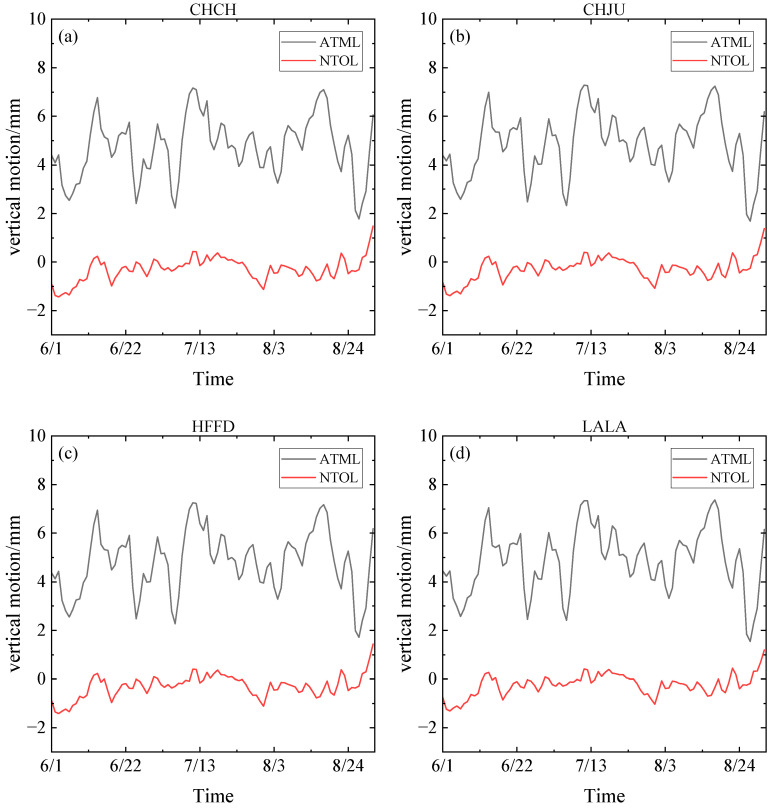
AHCORS atmospheric and non-tidal oceanic loads on vertical land deformation. (**a**) CHCH station; (**b**) CHJU station; (**c**) HFFD station; (**d**) LALA station.

According to the atmospheric and non-tidal oceanic load models provided by IMLS, the data was calculated and processed. In order to better observe the changes in the environmental load-induced deformation data during the whole study period, the vertical deformation map of atmospheric and non-tidal oceanic loads at the AHCORS station was calculated. And the results of each station are shown in Figure 4. Some conclusions can be drawn as follows:

The black curve in Figure 4 shows the vertical deformation map of the atmospheric load during the rainstorm. The vertical deformation time series of four CORS stations distributed around the Chaohu Lake area all show the same trend. During June, the atmospheric load caused land to rise gradually and following fall back. With the arrival of the rainy season, the land subsidence reached the maximum value around 15 July. The average value of land subsidence of the four stations in this period rose 2.4 mm compared with that on 1 June. The land surface load caused by atmospheric load began to fall gradually after the rainstorm discharge. After 5 August, the land surface deformation of each station rose to the normal value and then fluctuated within a certain range.

The red curve in Figure 4 shows the vertical deformation map of the non-tidal oceanic load during the rainstorm, and the four CORS stations distributed around the Chaohu Lake area all show the same trend. The non-tidal oceanic load first showed a steady and slow rising trend, and reached the peak on 12 July during the rainstorm period. The average value of the deformation at each station rose by 1.3 mm compared with that on 1 June, and then gradually fell back and fluctuated within a certain range.

### 3.2. Effect of Hydrological Load on Vertical Land Deformation

**Figure 5:**
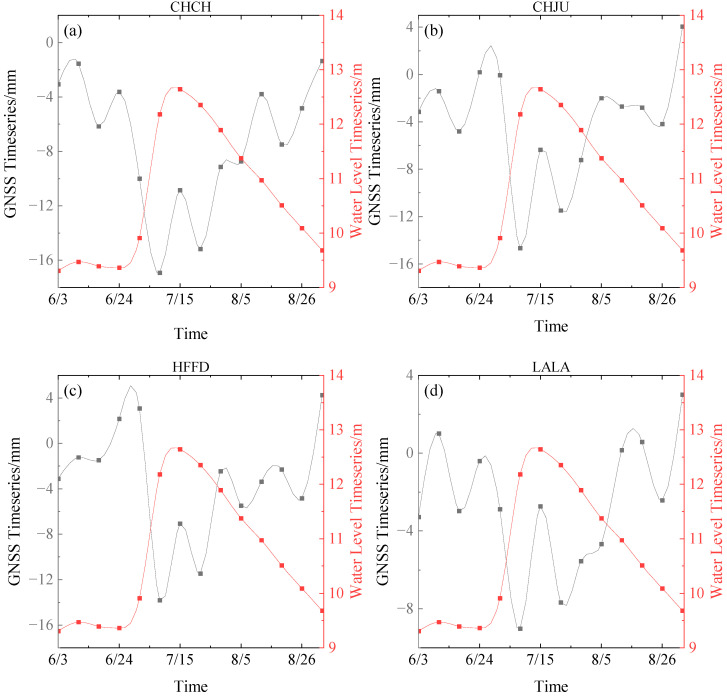
Correlation analysis between GNSS time series and Water Level time series at different stations. (**a**) CHCH station; (**b**) CHJU station; (**c**) HFFD station; (**d**) LALA station.

In order to obtain the vertical displacement caused by hydrological factors, the vertical displacement correction data of the IMLS institution was deducted from the GNSS time series. Then, an accurate analysis of the spatial-temporal dynamic evolution of surface deformation driven by hydrological signals around the Chaohu Lake area was carried out. The correlation between the water level data and the vertical displacement time series of the CORS sites was calculated by using the Pearson correlation coefficient method. The results of the Pearson correlation coefficient method are shown in Figure 5 and Table 4. Temporal and spatial evolution can be shown as follows.

Temporal dynamic evolution: As can be seen from Figure 5, before June 20, the water level of Chaohu Lake had no obvious fluctuation, and the vertical displacement of each CORS station had a small change. Between June 20 and July 10, affected by the heavy rainfall and the flood discharge capacity of Chaohu Lake, the water level of Chaohu Lake showed a rapidly rising trend, and it was found that the vertical displacements of the CORS stations had decreased significantly. Compared with June 10, the vertical displacement of the nearest station (CHCH) to Chaohu Lake decreased by 15.38 mm, CHJU station and HFFD station decreased by 13.25 mm and 12.59 mm respectively. The vertical displacement of the farthest station (LALA) to Chaohu Lake was decreased by 10.02 mm. After July 10, affected by the reduction of rainfall and water conservancy project scheduling, the water level of Chaohu Lake gradually declined and tended to restore the original level before the rainstorm, all CORS sites showed uplifting trends of different degrees. Compared with the occurrence of heavy rainfall before, the surface was slightly uplifted and confirmed the elastic deformation principle of disc load.

Spatial dynamic evolution: As can be seen from Table 4, the correlation coefficients between GNSS data and hydrological signals during heavy rainfall were computed, and it was found that GNSS time series had a certain correlation with water level time series. All the correlation coefficients were strongly negative. The correlation coefficients of CHCH station, which was the nearest to Chaohu Lake, was −0.8, and LALA station, which was the farthest to Chaohu Lake, was also as high as −0.64. The response of water level change to the displacement of GNSS stations was fairly obvious, this phenomenon confirmed the elastic deformation principle of disc load.

### 3.3. Validation of the Effect of Hydrologic Load on Vertical Land Deformation

**Figure 6:**
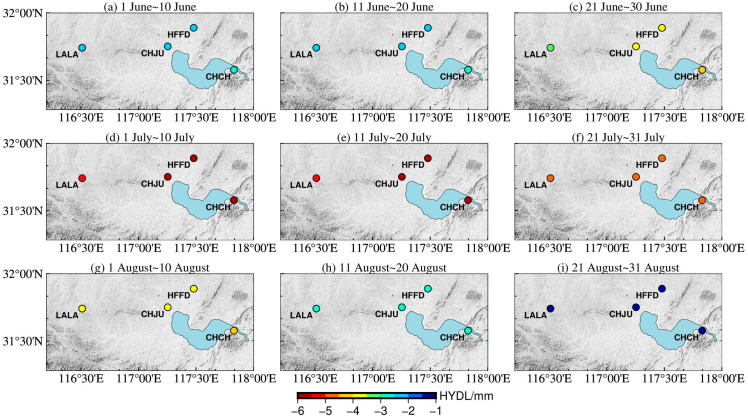
Vertical deformation of hydrological load for IMLS Institution during 1 June~31 August. (**a**) 1 June~10 June; (**b**) 11 June~20 June; (**c**) 21 June~30 June; (**d**) 1 July~10 July; (**e**) 11 July~20 July; (**f**) 21 July~31 July; (**g**) 1 August~10 August; (**h**) 11 August~20 August; (**i**) 21 August~31 August.

**Figure 7:**
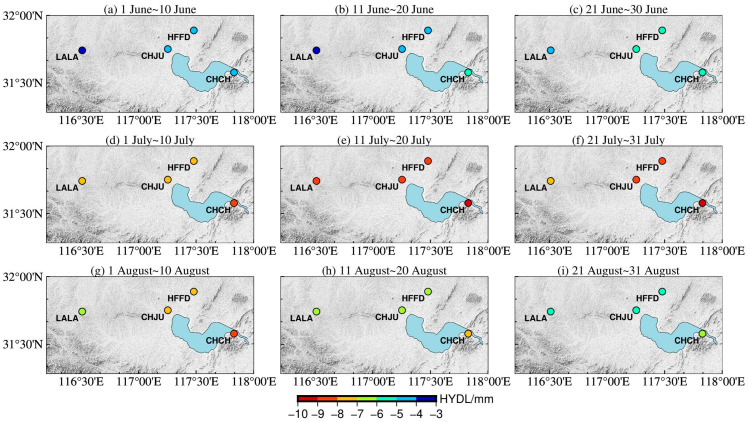
Vertical deformation of hydrological load for GFZ Institution during 1 June~31 August. (**a**) 1 June~10 June; (**b**) 11 June~20 June; (**c**) 21 June~30 June; (**d**) 1 July~10 July; (**e**) 11 July~20 July; (**f**) 21 July~31 July; (**g**) 1 August~10 August; (**h**) 11 August~20 August; (**i**) 21 August~31 August.

**Figure 8:**
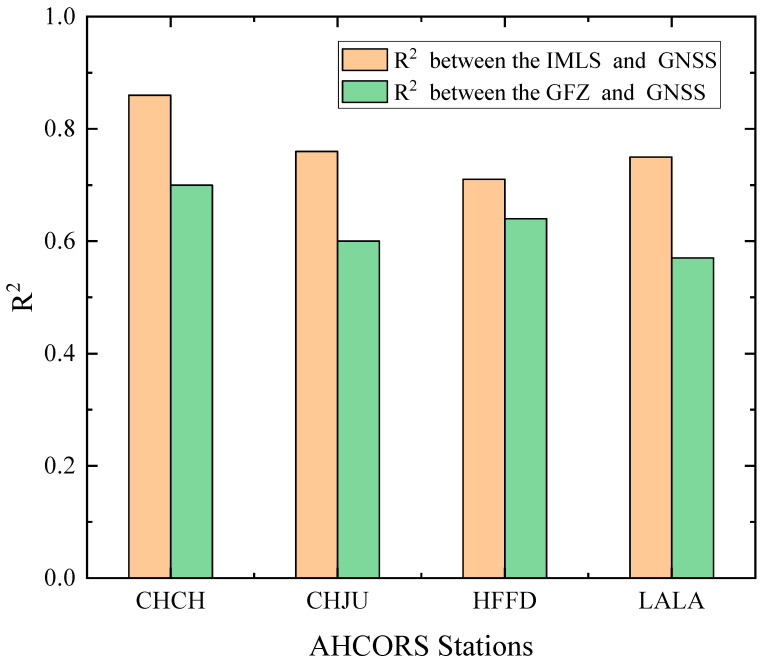
R^2^ between the different model data and the GNSS monitoring results.

In order to further verify the spatial-temporal dynamic evolution of surface deformation driven by the hydrological signals around Chaohu Lake, the hydrological load-induced deformation data obtained by the IMLS and GFZ institutions were quantitatively analyzed for nine time periods. The results are shown in Figure 6 and Figure 7, respectively. The consistency between the different institutional models and the GNSS results of the CORS sites was calculated by using the Coefficient of determination method. The results of the Coefficient of determination method are shown in Figure 8.

Hydrological load analysis by IMLS: Figure 6a,b showed that the vertical deformation of hydrological load at each CORS station changed slightly during the first 20 days of the main storm season around Chaohu Lake; Figure 6c–e showed that the CORS stations sank rapidly due to the influence of the rapid rise of the water level. Among them, the land subsidence of the closest station (CHCH) was the most obvious, and the land subsidence of the furthest station (LALA) was the smallest; Figure 6f–i indicated that the stations began to rise slowly and were higher compared to June 1 due to the influence of the falling water level. During heavy rainfall, combined with Figure 8, the average value of the Coefficient of determination between the IMLS model data and the GNSS monitoring results was 0.77, so the vertical deformation induced by hydrological load of IMLS was generally consistent with GNSS monitoring results.

Hydrological load analysis by GFZ: Figure 7a,b showed that the vertical deformation of hydrological load at each CORS station changed slightly during the first 20 days of the main storm season around Chaohu Lake; Figure 7c–f showed that the CORS stations sank rapidly due to the influence of the rapid rise of the water level. Among them, the land subsidence of the closest station (CHCH) was the most obvious, and the land subsidence of the furthest station (LALA) was the smallest; Figure 7g–i indicated that the stations began to rise slowly and were lower compared to June 1 due to the influence of the falling water level. During heavy rainfall, combined with Figure 8, the average value of the Coefficient of determination between the GFZ model data and the GNSS monitoring results was 0.63, so the vertical deformation induced by hydrological load of GFZ was generally consistent with GNSS monitoring results.

## 4. Discussion

Previous studies have demonstrated that GRACE satellite acquires trends and seasonal features of terrestrial water storage with unprecedented accuracy. However, the rough observation of the GRACE satellite is negative in detecting changes in terrestrial water in a small-scale region. The study area of this paper is small and the GRACE method is not applicable. Therefore, the hydrological data in this paper was water level and flow data, which was obtained from the Hefei Hydrological Comprehensive Service System. At the same time, model deformation data was provided from IMLS and GFZ to verify the GNSS results and increase the reliability of the paper.

In Section 3, during the rainstorm period, compared with the non-tidal oceanic load, the overall vertical displacement of the surface caused by the atmospheric load was larger, and the two effects on the surface deformation had opposite trends. Atmospheric load acted as a lifting effect on the surface, while non-tidal oceanic loads acted as a suppression effect. Meanwhile, the response of water level change to the displacement of GNSS stations was fairly obvious, this phenomenon confirmed the elastic deformation principle of disc load. Combined with the Pearson correlation coefficient method, the rise of water level will lead to the downward vertical movement of CORS stations. The results indicated that the change of groundwater level played a major role in the change of land surface elevation around the Chaohu Lake area. Furthermore, Figure 6 and Figure 7 showed that the modeled deformations were smaller than the GPS observations results. Since the short-term heavy rainfall mainly affects surface water [42], the load-induced deformation data provided by GNSS, which includes the groundwater [43,44], makes the vertical deformation of hydrological load larger, and the period of vertical deformation longer. Combined with the Coefficient of determination method, the deformation trends of IMLS and GFZ were generally consistent with the GNSS monitoring results, and GNSS was more suitable for the study of hydrological load deformation of the lakes during the heavy rainfall period.

The spatial migration of terrestrial water storage causes elastic hydrologic loading deformation on the surface, and if that non-tectonic deformation can be estimated, it will be significant to our understanding of crustal deformation and tectonic movements. This paper utilized multi-source data and relative analysis methods to provide scientific results for the spatial-temporal dynamic evolution of land deformation driven by hydrological signals around Chaohu Lake. This study is of great significance for understanding the correlation between water resources and GNSS vertical displacement in the Chaohu Lake area. It is helpful to strengthen the management of groundwater resources in the Chaohu area, rationally exploit and utilize water resources, and take corresponding measures to maintain the infrastructure timely to reduce the impacts of land subsidence. At the same time, the study provides some references for agricultural irrigation, environmental governance, urban planning and other fields to promote the sustainable development of the regions. In this paper, only the vertical land deformation is studied, the land deformation caused by heavy rain in the Chaohu Lake area also has a certain influence on the horizontal direction. In the future, it is necessary to conduct a deeper exploration of the land deformation with the horizontal displacement. In addition, surface subsidence will be analyzed in combination with rainfall, temperature and other data in the study to provide useful information for disaster monitoring and forecasting in the Chaohu Lake area.

## 5. Conclusions

In this paper, the observation files of AHCORS station from June 2016 to August 2016 provided by Anhui Provincial Bureau of Surveying and Mapping, Hefei Hydrological Comprehensive Service System obtained the hydrological data of Chaohu Lake during the same period, and the environmental load-induced deformation model data of IMLS and GFZ institutions to jointly analyze the spatial-temporal dynamic evolution of the land deformation of Chaohu Lake driven by hydrological signals during the rainstorm.

During the heavy rainfall, the rapidly increasing hydrological load around Chaohu Lake region caused the CORS stations near Chaohu Lake to have greater deformation than the more distant ones, with the vertical displacement of the nearest CHCH station decreasing by 15.38 mm and that of the furthest LALA station decreasing by 10.02 mm within one month. Pearson’s correlation coefficients with the water level were as high as −0.80 and −0.64, respectively, which revealed the spatial-temporal dynamic evolution of land deformation around Chaohu Lake and confirmed the elastic deformation principle of disc load. The deformation models can reflect the vertical deformation caused by the surface hydrological load of Chaohu Lake under the changing environment, there were numerical differences due to the existence of systematic bias between the different models, but the trends were generally consistent with the GNSS monitoring results. And the GNSS was more effective in monitoring the short-term hydrological load compared with the deformation models.

Heavy rainfall can cause serious damage to the ground surface, and this study analyzes the water level change and vertical deformation of surface hydrological load around Chaohu Lake during heavy rainfall. The results reveal the spatial-temporal dynamic evolution of the land deformation, which will provide a certain reference value for the future investigation of land deformation driven by hydrological signals around Chaohu Lake.

## Figures and Tables

**Table 1:** GAMIT data processing strategy.

Data Processing Strategy	Options
Sampling interval	set sint = 30
Number of epochs	set nepc = 2880
Choice of Experiment	RELAX.
Choice of Observable	LC_AUTCLN
Zenith Delay Estimation	Y
Type of Analysis	1-ITER
DMap	VMF1
WMap	VMF1
Use map.grid	Y
Use atml.grid	Y
Use otl.grid	Y

**Table 2:** Data product information provided by GFZ and IMLS.

Institution		IMLS		GFZ
Data Type	ATML	NTOL	HYDL	HYDL
Physical Model	MERRA2	MPIOM06	MERRA2	LSDM
Spatial resolution	2′ × 2′	2′ × 2′	2′ × 2′	0.5° × 0.5°
Temporal resolution	6 h	3 h	6 h	24 h

**Table 3:** The value range of Pearson correlation coefficient.

PCC	Degree of Correlation
−0.2~0/0~0.2	No correlation
−0.4~−0.2/0.2~0.4	Low correlation
−0.6~−0.4/0.4~0.6	Medium correlation
−0.8~−0.6/0.6~0.8	Relatively high correlation
−1~−0.8/0.8~1	High correlation

**Table 4:** Pearson correlation coefficient between water level change and vertical displacement change at different stations.

CHCH	CHJU	HFFD	LALA
−0.80	−0.72	−0.76	−0.64
